# Quantitative Proteomics Analysis of Tissue Interstitial Fluid for Identification of Novel Serum Candidate Diagnostic Marker for Hepatocellular Carcinoma

**DOI:** 10.1038/srep26499

**Published:** 2016-05-24

**Authors:** Wei Sun, Baocai Xing, Lihai Guo, Zhilei Liu, Jinsong Mu, Longqin Sun, Handong Wei, Xiaohang Zhao, Xiaohong Qian, Ying Jiang, Fuchu He

**Affiliations:** 1 State Key Laboratory of Proteomics, Beijing Proteome Research Center, National Center for Protein Sciences Beijing, Beijing Institute of Radiation Medicine, Beijing 102206, China; 2 Beijing Cancer Hospital, Beijing 100036, China; 3 AB SCIEX, Beijing 100015, China; 4 MOE Key Laboratory of Bioinformatics, School of Life Sciences, Tsinghua University, Beijing 100084, China; 5 302 Hospital of PLA, Beijing 100039, China; 6 State Key Laboratory of Molecular Oncology, Cancer Institute & Hospital, Chinese Academy of Medical Sciences & Peking Union Medical College, Beijing 100021, China; 7 Institutes of Biomedical Sciences, Fudan University, Shanghai 200032, China

## Abstract

Hepatocellular carcinoma (HCC) is the fifth most common malignant cancer in the world. The sensitivity of alpha-fetoprotein (AFP) is still inadequate for HCC diagnosis. Tissue interstitial fluid (TIF), as the liquid microenvironment of cancer cells, was used for biomarker discovery in this study. Paired tumor and nontumor TIF samples from 6 HBV-HCC patients were analyzed by a proteomic technique named iTRAQ (isobaric tag for relative and absolute quantitation). Totally, 241 up-regulated proteins (ratio ≥ 1.3, *p* < 0.05) and 288 down-regulated proteins (ratio ≤ −1.3, *p* < 0.05) in tumor TIF were identified. Interestingly, proteins in S100 family were found remarkably up-regulated in tumor TIF. One dramatically up-regulated protein S100A9 (ratio = 19) was further validated by ELISA in sera from liver cirrhosis (LC, HCC high risk population) and HCC patients (n = 47 for each group). The level of this protein was significantly elevated in HCC sera compared with LC (*p* < 0.0001). The area under the curve of this protein to distinguish HCC from LC was 0.83, with sensitivity of 91% (higher than AFP) and specificity of 66%. This result demonstrated the potential of S100A9 as a candidate HCC diagnostic biomarker. And TIF was a kind of promising material to identify candidate tumor biomarkers that could be detected in serum.

Hepatocellular carcinoma (HCC) is one of the most malignant tumors in the world, with an average life expectancy of less than 12 months after diagnosis of clinically apparent HCC^1^. Early diagnosis of HCC, especially in the high-risk populations with chronic liver diseases, is essential for the effective treatment. Liver cirrhosis (LC) underlies HCC in approximately 80–90% of cases worldwide^2^. In China and other South East Asian countries with high prevalence of hepatitis B virus (HBV) infection, HBV-associated HCC arises almost exclusively in patients with cirrhosis^3^. Cirrhotic patients with HBV background become the major high-risk population of HCC in these countries. Though AFP (alpha-fetaprotein) and ultrasonography are widely used for HCC screening in high-risk populations, AFP is not sufficient to distinguish HCC from LC with a sensitivity of 39~65%, a specificity of 76~94%, and a positive predictive value of 9~50%^4^.

Proteomics has been applied for the discovery of novel HCC serum diagnostic markers. Previous works mainly focused on plasma/serum or tissue samples. However, the existence of high-abundance proteins and the large dynamic range of serum proteins is the major challenge for identification of serum proteins with low abundance in the discovery phase^5^. Moreover, the alterations in serum may be related to other concomitant disorders besides tumor iteself. Though tissues could present proteome changes more specific to tumor, the potential for clinical application will be limited if the proteins could not be secreted into the blood. And few studies performed further validation of tissue proteins in serum samples.

Following Celis’ innovative work on tissue interstitial fluid (TIF)^6^, our group for the first time analyzed the proteome of liver TIF, which is the interface between circulating body fluids and liver intracellular fluid^7^. In this study, we collected paired tumor and nontumor liver TIFs from HCC patients with cirrhosis to discover tumor originated proteins that could be transported into peripheral blood. Enzyme linked immunosorbent assay (ELISA) validation of up-regulated protein in LC and HCC sera demonstrated that TIF could be used for identification of candidate tumor biomarkers that could be detected in serum.

## Results

### Proteins identified in HCC TIF samples

**Figure 1:**
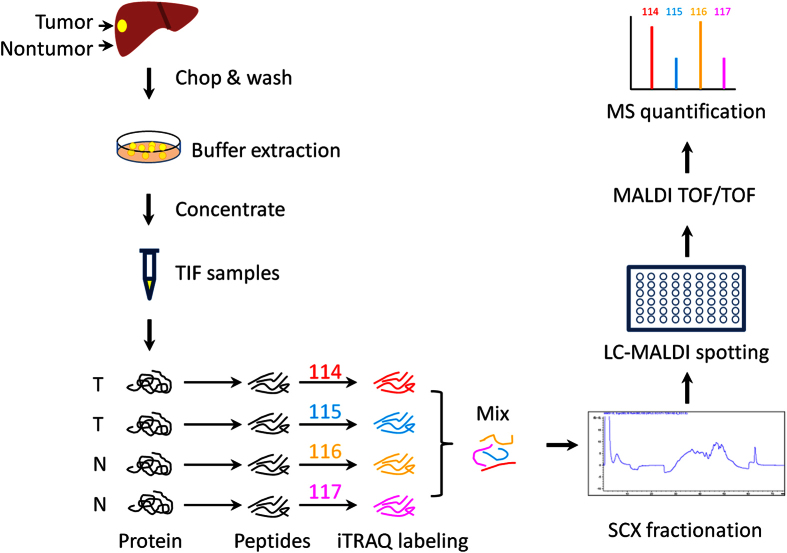
Diagram for preparation and iTRAQ analysis of liver TIF samples from HCC tissues.

**Figure 2:**
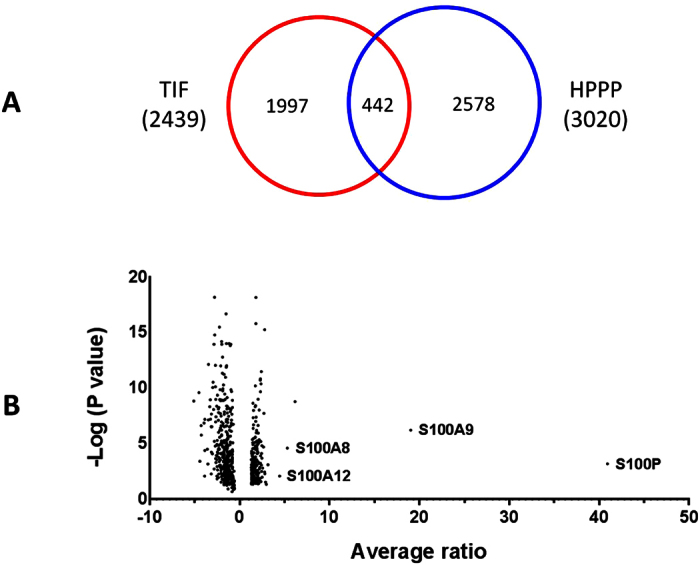
Proteins identified and differentially expressed in HCC TIFs. (**A**) Proteins identified in HCC TIF and HPPP. (**B**) Volcano plot of differentially expressed proteins.

The paired tumor and nontumor TIF samples from 6 HBV-HCC patients were analyzed by iTRAQ (Fig. 1). Totally, 2439 proteins were identified (protein FDR < 0.01 and ≥2 unique peptides with FDR < 0.05, Table S1), including 442 proteins (18%) which could be detected by Human Plasma Proteome Project (HPPP, http://www.hupo.org/research/hppp/) (Fig. 2A). The iTRAQ data has been submitted to iProx (http://www.iprox.org).

### iTRAQ quantification of differentially expressed proteins in HCC TIF samples

**Figure 3:**
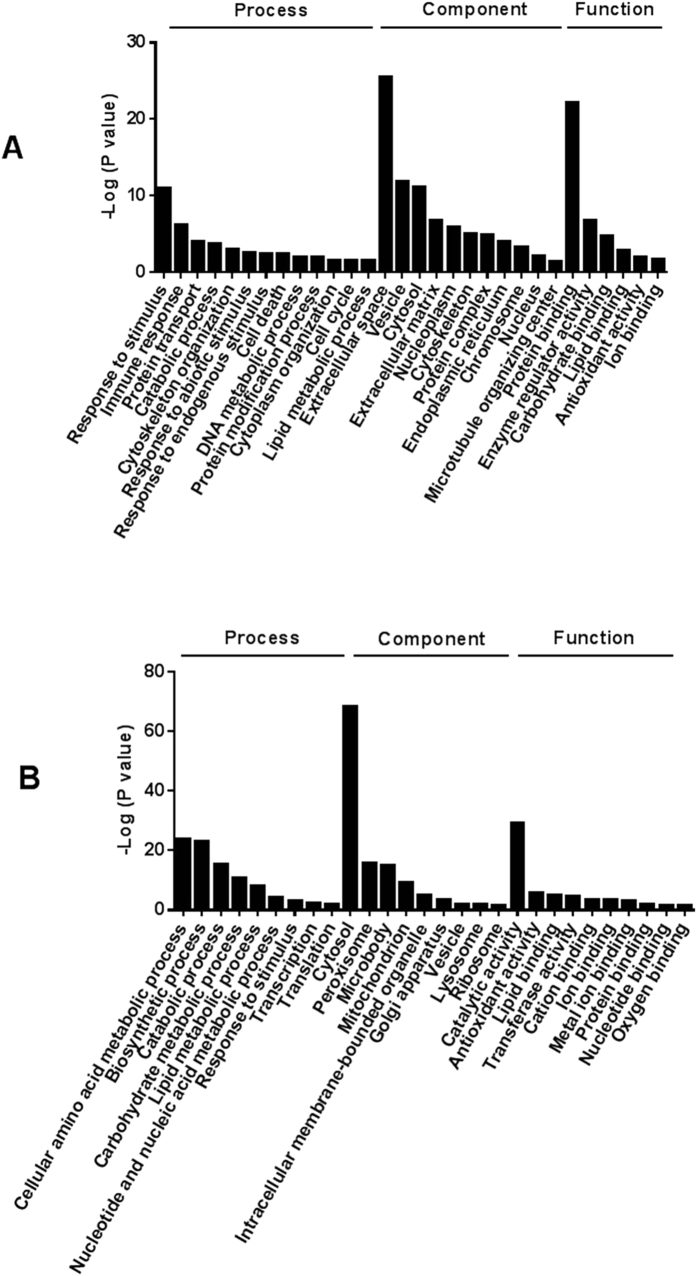
Significantly enriched (*p* < 0.05) Gene Ontology Terms in up-regulated (**A**) and down-regulated (**B**) proteins.

Among the 2428 proteins which have quantification information, 241 proteins were up-regulated (ratio_tumor/nontumor_ ≥ 1.3, *p* < 0.05) and 288 proteins were down-regulated (ratio_tumor/nontumor_ ≤ −1.3, *p* < 0.05) in tumor TIF (Table S1, Fig. 2B). Gene Ontology analysis showed that proteins involved in response to stimulus, immune response, and proteins in extracellular space and vesicle were significantly enriched in up-regulated proteins, while proteins involved in cellular amino acid metabolic process and catalytic activity were significantly enriched in down-regulated proteins (Fig. 3).

According to Uniprot database annotation, 36 (15%) up-regulated proteins could be expressed by immune cells or organs in physiological condition (Table S2), including bone marrow, thymus, spleen and lymph node, or leukocytes/granulocytes (especially neutrophils), macrophages/monocytes, T- and B-lymphocytes, platelets myeloid cells and lymphoblasts (Figure S1). At the same time, 91 (32%) down-regulated proteins could be expressed in the liver, including 10 proteins specificly expressed in the liver (Table S3).

### Western blot validation of S100A9

**Figure 4:**
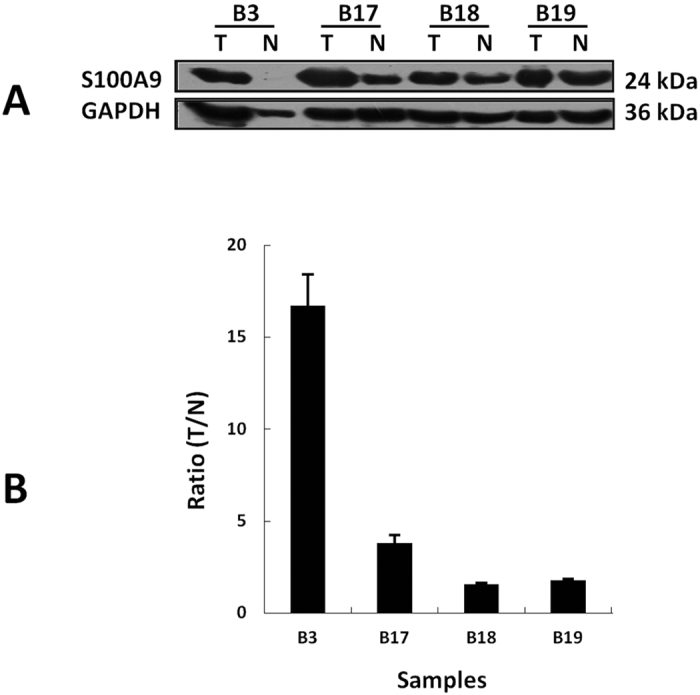
Western blot validated the up-regulation of S100A9 in HCC TIF samples. (**A**) Western blot of 4 paired HCC TIF samples. (**B**) Expression ratios of S100A9 in 4 paired samples (repeated three times).

The expression level of S100A9 was detected by Western blot in paired TIF samples from other 4 HCC patients (clinical information shown in Table 1). The up-regulation of S100A9 in tumor TIF was also found in these samples, especially with more than 15 folds upregulation in patient B3 (Fig. 4).

### ELISA validation of S100A9

**Figure 5:**
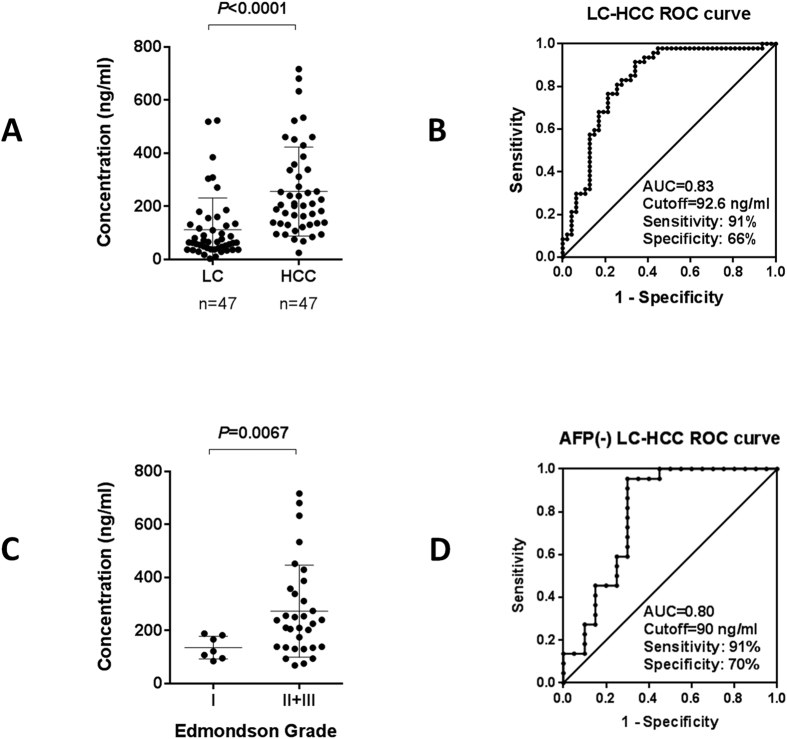
ELISA validation of serum S100A9 in LC and HCC patients. (**A**) S100A9 concentration in LC and HCC sera. (**B**) ROC curve of S100A9 to distinguish LC and HCC patients. (**C**) S100A9 concentration in well differentiated (Edmondson grade I) and less differentiated (Edmondson grade II+III) HCC sera. (**D**) ROC curve of S100A9 to distinguish AFP negative (≤20 ng/ml) LC and HCC patients.

ELISA validation for S100A9 was performed in 94 serum samples, including 47 HBV infected LC (39M/8F, age = 46 ± 9) and 47 HBV-HCC (40M/7F, age = 50 ± 11, 22 cases with AFP ≤ 20 ng/mL). Compared with LC patients (112 ± 119 ng/ml), S100A9 level significantly increased in HCC patients (256 ± 167 ng/ml) (*p* < 0.0001, shown in Fig. 5A). GraphPad Prism software was used for receiver operating characteristic (ROC) curve analysis. The area under the curve (AUC) of this protein to distinguish HCC from LC was 0.83 (95% confidence interval [CI]: 0.74–0.91). If the cutoff value was set to be 92.6 ng/ml, the sensitivity and specificity of S100A9 was 91% and 66%, respectively (Fig. 5B).

### Serum levels of S100A9 in well- and less-differentiated HCC cases

ELISA results of S100A9 in 47 HCC patients were analyzed according to Edmondson grade (7 cases grade I, 30 cases grade II and 2 cases grade III). The serum levels of S100A9 were significantly increased in less-differentiated (Edmondson grade II and III) cases than those in well-differentiated (Edmondson grade I) HCC cases (*p* = 0.0067, shown in Fig. 5C).

### Serum levels of S100A9 in AFP negative HCC cases

In samples with negative AFP (≤20 ng/ml), the AUC of S100A9 to distinguish HCC from LC was 0.80 (95% confidence interval [CI]: 0.65–0.94). If the cutoff value was set to be 90 ng/ml, the sensitivity and specificity of S100A9 was 91% and 70%, respectively (Fig. 5D).

## Discussions

In China, the LC patients with HBV infection backgroud is the major high-risk population of HCC. In order to find novel serum markers which could diagnose HCC in LC patients, we analyzed the paired tumor and nontumor TIF samples from 6 HBV-HCC patients with LC background. Totally, 2439 proteins were identified, among which 18% proteins could be detected by HPPP. This proportion is similar to Celis’ result in breast cancer TIFs (14%)^6^. And these HPPP identified proteins could be preferentially selected for validation in sera.

Using iTRAQ quantification method, 241 up-regulated and 288 down-regulated proteins were identified in HCC tumor TIF. Proteins involved in response to stimulus, immune response, and proteins in extracellular space and vesicle were significantly enriched in HCC up-regulated proteins, including previously reported proteins such as aldo-keto reductase family 1 member B10^8^, fibronectin^9^, protein disulfide-isomerase^10^, high mobility group protein B2^11^, 78 kDa glucose-regulated protein^12^, galectin-3^13^, and so on. According to Uniprot database annotation, 36 (15%) up-regulated proteins could be expressed by immune cells or organs in physiological condition (Table S2), indicating the involvement of immune response in cancer microenvironment during the process of HCC. Over 30% of up-regulated proteins could be detected in thymus, lymph node or bone marrow and over 50% of up-regulated proteins could be expressed in leukocytes (especially neutrophils) or macrophages/monocytes. It is reported that various immunocytes could infiltrate into the tumor and regulate the development and progression of HCC^14^. For example, leukocytes within HCC microenvironment stimulated hepatocellular ROS and telomere DNA damage. Inhibiting neutrophil accumulation in chronic liver disease may be a new therapeutic strategy in HCC^15^. High macrophage infiltration predicts poor prognosis in HCC patients^16^. Inhibition of monocytes/macrophages in liver markedly reduced tumor growth *in vivo*^17^. Our iTRAQ result of HCC TIFs demonstrated that the immune response was involved in HCC microenvironment and that this information could be detected by analysis of TIF.

At the same time, proteins involved in cellular amino acid metabolic process and catalytic activity were significantly enriched in down-regulated proteins. Among these proteins, 91 (32%) could be expressed in the liver, including 10 proteins specificly expressed in the liver (Table S3). This result indicated that the normal hepatic physiological functions have been lost in the dedifferentiated tumor tissues, as have been demonstrated in previous studies.

Interestingly, 4 of the top 5 up-regulated proteins in tumor TIF were all S100 family members, including S100P (ratio = 40.9), S100A9 (ratio = 19.0), S100A8 (ratio = 5.3) and S100A12 (ratio = 4.5). S100 proteins are a class of calcium binding family. It was reported that S100 proteins played significant roles in tumor progression and metastasis by modulating tumor microenvironment^18^. As intracellular Ca^2+^ sensors and extracellular factors, S100 proteins were found dysregulated in a variety of human cancers^19^.

Over-expression of S100P in HCC and other tumor tissues were observed and S100P was reported to be a prognostic factor for HCC early recurrence^20^. S100P-silencing suppressed HCC cell growth and inhibited cyclin D1 and CDK expression^21^. S100A8 and S100A9 are NF-kappa B target genes during malignant progression of inflammation-associated liver carcinogenesis^22^. The absence of S100A8/A9 could significantly reduce the tumor size in HCC model^23^. S100A8, as an endogenous ligand for toll-like receptor 4, contributes to lung metastasis^24^. S100A9 promotes HCC cell growth and invasion through RAGE-dependent MAPK signaling cascades^25^. S100A12 might be a potential prognostic marker for early recurrence of huge HCC after radical resection^26^.

The up-regulation of these S100 family members is also coincident with the up-regulation of leukocytes-derived proteins in HCC TIF. For example, S100A8, S100A9 and S100A12 are highly abundant proteins released by immune cells including neutrophils and monocytes. As components of neutrophil chemokine network with CXCL1 and CXCL2, S100A8 and S100A9 are associated with migration of myeloid derived suppressor cells in tumor^27^. On the contrary, mice lacking S100A9 expressed less neutrophil chemokines^15^.

Two of these four up-regulated S100 family members, S100A9 and S100A8 could be detected by HPPP. Because of the lack of qualified S100P antibodies, we chose S100A9 for further validation. Though up-regulation of S100A9 was observed in HCC tissues^28^, there is no report about S100A9 expression level in HCC serum samples. Using an ELISA kit with paired capture and detection antibodies, we detected the serum levels of S100A9 in 47 LC and 47 HCC patients. The result showed that S100A9 level was significantly elevated in HCC sera (256 ± 167 ng/ml) compared with LC (112 ± 119 ng/ml) (*p* < 0.0001). ROC curve analysis showed that AUC was 0.83 to distinguish HCC from LC, with a sensitivity of 91% and a specificity of 66%. However, only 53% (25/47) HCC cases in this experiment had positive AFP levels (>20 ng/ml). This result indicated that S100A9 is a potential serum marker for diagnosis of HCC with a relatively higher sensitivity.

The expression levels of S100A9 in samples with negative AFP (≤20 ng/ml) were also analyzed. The ROC curve result showed that AUC was 0.83 to distinguish HCC from LC, with sensitivity of 91% and specificity of 70%. That means S100A9 could also be used for diagnosis of AFP negative HCC.

Further analysis found that the serum levels of S100A9 in less-differentiated (Edmondson grade II and III) HCC cases were significantly higher than those in well-differentiated (Edmondson grade I) cases (*p* = 0.0067), indicating that serum S100A9 might be a potential indicator for less-differentiation in HCC.

In summary, using iTRAQ technique, we analyzed paired tumor and nontumor TIF samples from 6 HBV-HCC patients. One dramatically up-regulated protein S100A9 was further validated by ELISA in sera from 47 LC and 47 HCC patients. The AUC of S100A9 to distinguish HCC from LC was 0.83, with a sensitivity of 91% and a specificity of 66%. Our result showed that S100A9 could be a candidate HCC biomarker for further validation in larger populations. And TIF was a kind of promising material to identify candidate tumor biomarkers that could be detected in serum.

## Materials and Methods

### Patient specimens

Tumor and adjacent non-tumor tissues from 6 HCC patients (details shown in Table 1) were collected for TIF analysis. Serum samples from 47 LC patients and 47 HCC patients (details shown in Table S4) were included for ELISA validation. HCC tissue samples were collected from Beijing Cancer hospital. Serum samples were collected from Beijing Cancer hospital (HCC), Cancer Institute & Hospital of Chinese Academy of Medical Sciences (HCC), and 302 Hospital of PLA (LC). Informed consents were obtained and the access to human samples was carried out in accordance with the approved guidelines of the Ethics Committee. All experimental protocols were approved by Beijing Institute of Radiation Medicine. The diagnosis of HCC relied on histological analysis of liver surgical resections. LC diagnosis was based on hepatic encephalopathy, ascites, bilirubin, albumin, prothrombin time and B-ultrasonic examination. All patients included in this study had only HBV infection background.

### Liver TIF samples preparation

Liver TIF samples were collected according to the previous report^7^. Briefly, fresh tissues were cut into small pieces in PBS containing protease inhibitor cocktail. Then tissue pieces were incubated at 37 °C in a CO_2_ incubator for 1 h after PBS washing. After centrifugation at 1000 g for 3 min, the supernatant was transferred to fresh Eppendorf tubes using a Paster pipet. Then samples were centrifuged at 2000 g for 8 min and then 20 000 g for 30 min at 4 °C. After concentration in a vacuum dehydrator for 1 h, the TIF samples were snap-frozen in liquid nitrogen and stored at −80 °C. No additional freeze-thaw was carried out before use.

### Treatment of serum samples

BD Vacutainer (no additive, BD Biosciences, Franklin Lakes, NJ, USA) was used to collect peripheral blood from patients in the morning before treatment. After collection, blood specimens were centrifuged at 2000 g for 5 min and then 13 500 g for 15 min at 4 °C within 1 hour of collection. All samples were stored at −80 °C until use.

### iTRAQ analysis of TIF samples

The TIF samples from the tumor or non-tumor tissues (30 μg each) were pooled, respectively. The 180 μg of pooled tumor or non-tumor TIF samples were separated into 4 tubes (90 μg each) and the pH was adjusted to 8.5 by adding 25 μl of TEAB buffer. Then samples were treated with 20 mM dithiothreitol at 56 °C for 60 min, 50 mM iodoacetamide in dark for 30 min. After digestion with 3 μg of trypsin (sequencing grade, Promega, France) at 37 °C overnight, the peptides were labeled with iTRAQ tags and dried, then redissolved in 0.5% trifluoroacetic acid and separated by Strong Cation Exchange (SCX) chromatography. SCX was carried out on a Polysulfoethyl 4.6 × 100 mm column (5 μm, 200 Å, PolyLC Inc, Maryland, USA). The peptides were eluted at the 45 min gradient from 100% buffer A (10 mM KH_2_PO_4_ pH 3.0, 25% acetonitrile) to 45% buffer B (10 mM KH_2_PO_4_ pH 3.0, 500 mM KCl, 25% acetonitrile) at the flow rate of 800 μL/min on Agilent 1210 LC system. Totally, 28 fractions were collected and dried. After being redissolved in 30 μl of 0.1% trifluoroacetic acid, each fraction was separated and spotted using the Tempo^TM^ LC-MALDI Spotting System (AB SCIEX, USA). Peptides were separated by a C18 AQ 150 × 0.2 mm column (3 μm, Michrom, USA) using a linear gradient formed by buffer A (2% acetonitrile, 0.1% trifluoroacetic acid) and buffer B (98% acetonitrile, 0.1% trifluoroacetic acid), from 5% to 35% of buffer B over 90 minutes at a flow rate of 2 μL/min. The eluted peptides were mixed with matrix solution (5 mg/mL in 70% acetonitrile, 0.1% trifluoroacetic acid) at a flow rate of 2 μL/min pushed by additional syringe pump. For each SCX fraction, 616 spots were spotted on a 123 × 81 mm LC-MALDI plate insert. Then the spots were analyzed using MALDI-TOF/TOF 5800 mass spectrometer (AB SCIEX, USA). A full-scan MS experiment (m/z range from 800 to 4000) was acquired, followed by MS/MS on the top 40 ions detected.

Relative quantification and protein identification were performed with the ProteinPilot^TM^ software (version 4.0.1; AB SCIEX). Each MS/MS spectrum was searched against a database (20100712 released UniProtKB/Swiss-Prot human database, 20282 entries) and a decoy database for FDR analysis (programmed in the software). The search parameters were as follows: trypsin enzyme; maximum allowed missed cleavages 1; iTRAQ 4plex mode; cysteine modification by methyl methane-thiosulfonate; biological modifications programmed in the algorithm. Proteins with unused Protein Score ≥ 2 (FDR < 0.01) and matching reliable peptides (≥2 unique peptides with FDR < 0.05) were considered as positively identified proteins.

Peptide abundances were calculated based on the areas of the monoisotopic peaks. Protein ratios were the average ratios of all quantified peptides. Proteins with quantification *P* value < 0.05 in at least two pairs (114:116, 114:117, 115:116, 115:117) and with the ratio > 1.3 (the average ratio of two repeat experiments) were considered as differentially expressed proteins, using a cutoff of 2 times standard deviation^29^.

### Western blot analysis

For each sample, 25 μg of protein was separated by a 12% SDS-PAGE gel and then electronically transferred onto a nitrocellulose membrane. After being blocked for 1 h at room temperature in TBST (20 mM Tris-HCl, 140 mM NaCl, pH 7.5, 0.05% Tween-20) containing 5% skim milk, the membrane was incubated with primary anti-S100A9 antibody (mouse monoclonal antibody, catalog ab24111, Abcam, Cambridge, USA, diluted 1:1,000) overnight at 4 °C. Then it was washed 3 times with TBST and incubated with horse-radish peroxidase-conjugated secondary antibody (diluted 1:5,000, Santa Cruz, CA, USA) for 1 h at room temperature. Visualization of the immunoreactive proteins was accomplished using enhanced chemiluminescence reagents (Pierce, Rockford, IL, USA).

### ELISA

S100A9 recombinant protein (catalog# H00006280-P01, Abnova) and matched antibody pair (catalog # H00006280-AP11, Abnova) were used for ELISA quantitative determination of serum S100A9 in 47 LC patients and 47 HCC patients, according to the manufacturer’s manual. In brief, a 96-well-plate was coated by the coating antibody over night followed by blocking for 2 h with blocking buffer. Then the diluted standard protein or serum samples (1:100) were added on the plate and incubated for 2 h. After adding the detection antibody for 1 h incubation, the secondary antibody was added and incubated for 1 h. The plate was washed 4 times with washing buffer after each step above. After 5 min incubation with substrate, the stop solution was added and the absorbance was read on a microwell plate reader at a wavelength of 450 nm within 10 min. The experiment was performed at room temperature and all the samples were assayed in duplicate.

## Additional Information

**How to cite this article**: Sun, W. *et al*. Quantitative Proteomics Analysis of Tissue Interstitial Fluid for Identification of Novel Serum Candidate Diagnostic Marker for Hepatocellular Carcinoma. *Sci. Rep*. **6**, 26499; doi: 10.1038/srep26499 (2016).

## Supplementary Material

Supplementary Information

Supplementary Table S1

## Figures and Tables

**Table 1:** Information of HCC patients analyzed by iTRAQ or Western blot.

Group	Patients No.	Sex	Age	HBV	Liver Cirrhosis	Edmondson Grade
iTRAQ	Y31	M	42	+	+	III
	Y67	M	49	+	+	III
	Y68	F	35	+	+	II
	Y76	M	61	+	+	III
	Y81	M	72	+	+	II
	Y110	M	54	+	+	III
Western blot	B3	M	62	+	−	II
	B17	M	58	+	+	II
	B18	F	37	+	+	I
	B19	M	72	+	+	II
