# Supplementary material for: Quantitative Proteomics Analysis of Tissue Interstitial Fluid for Identification of Novel Serum Candidate Diagnostic Marker for Hepatocellular Carcinoma
Source: Sci Rep. 2016 May 24;6:26499. doi: 10.1038/srep26499 (PMC4877711; doi:10.1038/srep26499)

**Title**: Quantitative Proteomics Analysis of Tissue Interstitial Fluid for Identification of Novel Serum Candidate Diagnostic Marker for Hepatocellular Carcinoma

**Authors**: Wei Sun, Baocai Xing, Lihai Guo, Zhilei Lui, Jinsong Mu, Longqin Sun, Handong Wei, Xiaohang Zhao, Xiaohong Qian, Ying Jiang, Fuchu He

**Supplementary Information: Table S2-S4, Figure S1.**

**Table S2. Up-regulated proteins which could be expressed by immune cells or organs.**

| **Entry** | **Protein names** | **Tissue specificity** |
| --- | --- | --- |
| O00148 | ATP-dependent RNA helicase DDX39A | Lower levels in thymus and spleen |
| P01009 | Alpha-1-antitrypsin | Leukocytes |
| P02788 | Lactotransferrin | Peripheral blood neutrophils |
| P04196 | Histidine-rich glycoprotein | Macrophages |
| P05109 | Protein S100-A8 | Myeloid cells |
| P05154 | Plasma serine protease inhibitor | Megakaryocytes and platelets. |
| P06396 | Gelsolin | Phagocytic cells and platelets |
| P06702 | Protein S100-A9 | Myeloid cells |
| P08670 | Vimentin | T- and B-lymphocytes |
| P0C0L5 | Complement C4-B | Lowest levels in the thymus and spleen |
| P11413 | Glucose-6-phosphate 1-dehydrogenase | Lymphoblasts and granulocytes |
| P13796 | Plastin-2 | Spleen and other lymph node-containing organs, peripheral blood T-lymphocytes, neutrophils, monocytes, B-lymphocytes, and myeloid cells |
| P14598 | Neutrophil cytosol factor 1 | Peripheral blood monocytes and neutrophils |
| P14780 | Matrix metalloproteinase-9 | Normal alveolar macrophages and granulocytes |
| P15144 | Aminopeptidase N (CD13) | Granulocytes and monocytes |
| P16949 | Stathmin | Thymus and bone marrow |
| P17931 | Galectin-3 | Activated macrophages |
| P25815 | Protein S100-P | Spleen and leukocyte |
| P26885 | Peptidyl-prolyl cis-trans isomerase FKBP2 | T-cells and thymus |
| P31146 | Coronin-1A | Thymus, spleen, bone marrow and lymph node |
| P33241 | Lymphocyte-specific protein 1 | Activated T-lymphocytes |
| P35579 | Myosin-9 | Leukocytes |
| P36222 | Chitinase-3-like protein 1 | Activated macrophages, articular chondrocytes, synovial cells |
| P40121 | Macrophage-capping protein | Macrophages and macrophage-like cells |
| P48595 | Serpin B10 | Myeloid cells and the bone marrow |
| P50552 | Vasodilator-stimulated phosphoprotein | Platelets |
| P63162 | Small nuclear ribonucleoprotein-associated protein N | Lymphoblasts |
| P67936 | Tropomyosin alpha-4 chain | Platelets |
| P80188 | Neutrophil gelatinase-associated lipocalin | Bone marrow |
| P80511 | Protein S100-A12 | Neutrophils, monocytes and activated macrophages |
| Q15084 | Protein disulfide-isomerase A6 | Platelets |
| Q16643 | Drebrin | Peripheral blood lymphocytes, including T-cells |
| Q6JBY9 | CapZ-interacting protein | Lymphoid organs, including spleen, thymus, peripheral blood leukocytes, lymph node and bone marrow |
| Q969H8 | Myeloid-derived growth factor(IL-25) | Bone marrow cells |
| Q96PD5 | N-acetylmuramoyl-L-alanine amidase | Lymph nodes and thymus |
| Q9UBG0 | C-type mannose receptor 2 (CD280) | Spleen, thymus and macrophages |

**Table S3. Down-regulated proteins which are expressed in the liver.**

| **Entry** | **Protein names** | **Expression in the liver** |
| --- | --- | --- |
| O00763 | Acetyl-CoA carboxylase 2 | Highest level |
| O14832 | Phytanoyl-CoA dioxygenase, peroxisomal | Expressed |
| O15020 | Spectrin beta chain, non-erythrocytic 2 | Highly expressed |
| O43704 | Sulfotransferase family cytosolic 1B member 1 | Highly expressed |
| O60610 | Protein diaphanous homolog 1 | Expressed |
| O75521 | Enoyl-CoA delta isomerase 2, mitochondrial | Abundant |
| O75891 | Cytosolic 10-formyltetrahydrofolate dehydrogenase ( | Highly expressed |
| O75936 | Gamma-butyrobetaine dioxygenase | Moderately expressed |
| O76054 | SEC14-like protein 2 | Strong expression |
| O95154 | Aflatoxin B1 aldehyde reductase member 3 | Expressed |
| O95810 | Serum deprivation-response protein | Lower level |
| P02794 | Ferritin heavy chain | Specific |
| P07108 | Acyl-CoA-binding protein | High level |
| P08263 | Glutathione S-transferase A1 | Specific |
| P09488 | Glutathione S-transferase Mu 1 | Specific |
| P14550 | Alcohol dehydrogenase | Highly expressed |
| P16930 | Fumarylacetoacetase | Mainly expressed |
| P17516 | Aldo-keto reductase family 1 member C | Specific |
| P18283 | Glutathione peroxidase 2 | Mostly in liver |
| P21549 | Serine--pyruvate aminotransferase | Specific |
| P22307 | Non-specific lipid-transfer protein | Mainly expressed |
| P24298 | Alanine aminotransferase 1 | Mainly expressed |
| P28332 | Alcohol dehydrogenase 6 | Mainly expressed |
| P30043 | Flavin reductase | Predominantly expressed |
| P30711 | Glutathione S-transferase theta-1 | Low level |
| P31327 | Carbamoyl-phosphate synthase | Primarily expressed |
| P32189 | Glycerol kinase | Highly expressed |
| P33121 | Long-chain-fatty-acid--CoA ligase 1 | Highly expressed |
| P35520 | Cystathionine beta-synthase | Strongly expressed |
| P35558 | Phosphoenolpyruvate carboxykinase, cytosolic | Mainly expressed |
| P35573 | Glycogen debranching enzyme | Expressed |
| P40261 | Nicotinamide N-methyltransferase | Predominantly expressed |
| P40763 | Signal transducer and activator of transcription 3 (Acute-phase response factor) | Expressed |
| P43490 | Nicotinamide phosphoribosyltransferase | Expressed in large amount |
| P46019 | Phosphorylase b kinase regulatory subunit alpha, liver isoform | Predominantly expressed |
| P49189 | 4-trimethylaminobutyraldehyde dehydrogenase | High expression |
| P49753 | Acyl-coenzyme A thioesterase 2, mitochondrial | Strongest expression |
| P49888 | Estrogen sulfotransferase | Expressed |
| P50053 | Ketohexokinase | Most abundant |
| P50225 | Sulfotransferase 1A1 | Expressed |
| P51659 | Peroxisomal multifunctional enzyme type 2 | Highest concentration |
| P51857 | 3-oxo-5-beta-steroid 4-dehydrogenase | Highly expressed in liver |
| P54868 | Hydroxymethylglutaryl-CoA synthase, mitochondrial | Expressed |
| P55263 | Adenosine kinase | Highest level |
| P62070 | Ras-related protein R-Ras2 | Moderate level |
| P78417 | Glutathione S-transferase omega-1 | Highest expression |
| P80404 | 4-aminobutyrate aminotransferase, mitochondrial | Highest concentration |
| Q00266 | S-adenosylmethionine synthase isoform type-1 | Specific |
| Q04446 | 1,4-alpha-glucan-branching enzyme | Highest level |
| Q06278 | Aldehyde oxidase | Abundant |
| Q06520 | Bile salt sulfotransferase | Expressed |
| Q08426 | Peroxisomal bifunctional enzyme | Expressed |
| Q13057 | Bifunctional coenzyme A synthase | Expressed |
| Q13228 | Selenium-binding protein 1 | Highly expressed |
| Q14032 | Bile acid-CoA:amino acid N-acyltransferase | Expressed |
| Q14117 | Dihydropyrimidinase | Mainly expressed |
| Q14353 | Guanidinoacetate N-methyltransferase | Specific |
| Q14449 | Growth factor receptor-bound protein 14 | High level |
| Q14749 | Glycine N-methyltransferase | Specific |
| Q14914 | Prostaglandin reductase 1 | High expression |
| Q15067 | Peroxisomal acyl-coenzyme A oxidase 1 | Expressed |
| Q16719 | Kynureninase | Highest level |
| Q16775 | Hydroxyacylglutathione hydrolase, mitochondrial | Mainly expressed |
| Q68CK6 | Acyl-coenzyme A synthetase ACSM2B, mitochondrial | Specific |
| Q7Z4W1 | L-xylulose reductase | Highly expressed |
| Q8NFW8 | N-acylneuraminate cytidylyltransferase | Expressed |
| Q93088 | Betaine--homocysteine S-methyltransferase 1 | Mainly expressed |
| Q93099 | Homogentisate 1,2-dioxygenase | Highest expression |
| Q96C11 | FGGY carbohydrate kinase domain-containing protein | Low level |
| Q96DG6 | Carboxymethylenebutenolidase homolog | Highest level |
| Q96IU4 | Alpha/beta hydrolase domain-containing protein 14B | Expressed |
| Q96KP4 | Cytosolic non-specific dipeptidase | High level |
| Q99424 | Peroxisomal acyl-coenzyme A oxidase 2 | Expressed |
| Q99497 | Protein deglycase DJ-1 | Highly expressed |
| Q9BV57 | 1,2-dihydroxy-3-keto-5-methylthiopentene dioxygenase | Expressed |
| Q9H2A2 | Aldehyde dehydrogenase family 8 member A1 | Highly expressed |
| Q9H2M3 | S-methylmethionine--homocysteine S-methyltransferase BHMT2 | Expressed |
| Q9HC38 | Glyoxalase domain-containing protein 4 | Expressed |
| Q9HCE6 | Rho guanine nucleotide exchange factor 10-like protein | Expressed |
| Q9NQ94 | APOBEC1 complementation factor | Highest level |
| Q9NQR4 | Omega-amidase NIT2 | Expressed |
| Q9NVD7 | Alpha-parvin | Highest level |
| Q9NZN3 | EH domain-containing protein 3 | Moderately expressed |
| Q9UBQ7 | Glyoxylate reductase/hydroxypyruvate reductase | Most abundantly expressed |
| Q9UJM8 | Hydroxyacid oxidase 1 | Specific |
| Q9UKK9 | ADP-sugar pyrophosphatase | Most abundant |
| Q9UKL6 | Phosphatidylcholine transfer protein | Highest expression |
| Q9UQB8 | Brain-specific angiogenesis inhibitor 1-associated protein 2 | Highest expression (a short isoform) |
| Q9Y223 | Bifunctional UDP-N-acetylglucosamine 2-epimerase/N-acetylmannosamine kinase | Highest expression |
| Q9Y2S2 | Lambda-crystallin homolog | Highest level |
| Q9Y617 | Phosphoserine aminotransferase | High level |

**Table S4. Clinical information of cases included for ELISA analysis.**

(1) LC samples (n=47). (2) HCC samples (n=47)

| **(1) LC samples** | | | | | | | | | | |
| --- | --- | --- | --- | --- | --- | --- | --- | --- | --- | --- |
| **No.** | **Sex** | **Age(y)** | **HBs-Ag** | **HBs-Ab** | **HBe-Ag** | **HBe-Ab** | **HBc-Ab** | **HCV** | **AFP (ng/ml)** | **S100A9**  **(ng/ml)** |
| A3 | M | 37 | + | - | - | + | + | - | 13 | 64.48 |
| L13 | M | 42 | + | - | - | - | + | - | 388 | 37.44 |
| L15 | F | 39 | + | - | + | - | + | - | 281 | 3.23 |
| L17 | M | 39 | + | - | - | - | + | - | 4 | 80.56 |
| L21 | M | 40 | + | - | - | - | + | - | 13 | 34.48 |
| L22 | M | 45 | + | - | - | + | + | - | 120 | 18.08 |
| L25 | M | 53 | + | - | - | - | + | - | 23 | 87.68 |
| L26 | M | 52 | + | - | - | - | + | - | 29 | 38.91 |
| L32 | M | 52 | + | - | + | - | + | - | 1062 | 57.45 |
| L33 | M | 46 | + | - | - | + | + | - | 27 | 63.32 |
| L34 | M | 47 | + | - | - | - | + | - | 108 | 90.97 |
| L35 | M | 58 | + | - | - | - | + | - | 5 | 63.57 |
| L36 | M | 39 | + | - | - | - | + | - | 33 | 36.80 |
| L39 | F | 54 | + | - | + | - | + | - | 30 | 62.47 |
| L40 | M | 31 | + | - | + | - | + | - | 8 | 42.20 |
| L41 | M | 46 | + | - | + | - | + | - | 2 | 38.09 |
| L42 | M | 46 | + | - | + | - | + | - | 10 | 72.88 |
| L44 | F | 45 | + | - | - | + | + | - | 8 | 29.70 |
| L45 | F | 47 | + | - | - | + | + | - | 4 | 523.34 |
| L47 | M | 42 | + | - | - | + | + | - | 71210 | 35.62 |
| L48 | M | 50 | + | - | - | - | + | - | \ | 58.75 |
| L52 | M | 50 | + | - | - | + | + | - | \ | 48.22 |
| L53 | M | 36 | + | - | - | - | + | - | 2 | 37.26 |
| L54 | M | 39 | + | - | + | - | + | - | \ | 117.27 |
| L88 | M | 44 | + | - | + | - | + | - | 33 | 66.31 |
| L90 | M | 53 | + | - | + | - | + | - | 20 | 156.11 |
| L96 | M | 42 | + | - | + | - | + | - | 66 | 134.79 |
| L97 | M | 39 | + | - | + | - | + | - | 20 | 186.20 |
| L98 | F | 41 | + | - | + | - | + | - | 5 | 77.11 |
| L99 | F | 66 | + | - | + | - | + | - | 204 | 309.09 |
| L101 | F | 50 | + | - | - | - | + | - | 78 | 49.88 |
| L102 | M | 50 | + | - | - | - | + | - | 30 | 127.27 |
| L103 | M | 43 | + | - | + | - | + | - | 20 | 52.31 |
| L104 | M | 42 | + | - | + | - | + | - | 66 | 131.52 |
| A2 | M | 44 | + | - | - | + | + | - | 59 | 160.74 |
| B11 | M | 43 | + | - | - | + | + | - | 651 | 54.75 |
| B45 | M | 47 | + | - | - | - | + | - | 90 | 110.10 |
| B92 | M | 71 | + | + | - | + | + | - | 22 | 384.95 |
| B93 | M | 36 | + | - | + | - | + | - | 10 | 180.11 |
| B109 | M | 53 | + | - | - | + | + | - | 12 | 66.30 |
| B112 | M | 44 | + | - | - | + | + | - | 81 | 271.30 |
| B149 | M | 40 | + | - | + | - | + | - | 7 | 29.20 |
| B155 | F | 45 | + | - | + | - | + | - | 15 | 304.69 |
| B168 | M | 55 | + | - | - | + | + | - | 15 | 10.95 |
| B172 | M | 35 | + | - | + | + | + | - | 77 | 43.89 |
| B181 | M | 74 | + | - | + | - | + | - | 546 | 98.09 |
| B183 | M | 42 | + | - | - | + | + | - | 4 | 518.88 |

| **(2) HCC samples** | | | | | | | | | | | | | | |
| --- | --- | --- | --- | --- | --- | --- | --- | --- | --- | --- | --- | --- | --- | --- |
| **No.** | **Sex** | **Age(y)** | **Edmondson** | **AFP(ng/ml)** | **HBs-Ag** | **HBs-Ab** | **HBe-Ag** | **HBe-Ab** | **HBc-Ab** | **HCV** | **Cirrhosis** | **UICC'sTNM** | **TumorSize(cm)** | **S100A9**  **(ng/ml)** |
| C40 | M | 42 | III | 17178 | + | - | - | + | + | - | + | T1N0M0 | 3×4 | 633.49 |
| C464 | M | 54 | III | 3 | + | - | - | + | + | - | + | T2N0M0 | 5×4.2×6 | 175.36 |
| X32 | M | 40 | II | 5 | + | - | + | - | + | - | + | \ | 1.5×1.5×1.5 | 139.19 |
| Y10 | M | 59 | II | 3 | + | - | - | + | + | - | + | T2N0M0 | 6×7×6 | 135.65 |
| Y100 | M | 51 | II | 2 | + | - | - | + | + | - | + | T2N0M0 | 2.5×2 | 681.49 |
| Y102 | M | 53 | II | 8 | + | - | - | - | + | \ | + | T1N0M0 | 3×2.5 | 240.75 |
| Y105 | M | 42 | I | 10 | + | - | + | - | + | - | + | T1N0M0 | 4×4 | 188.71 |
| Y107 | M | 36 | II | 1029 | + | - | + | - | + | - | + | T1N0M0 | 2.2×3 | 338.55 |
| Y14 | M | 44 | \ | 14030 | + | - | + | - | + | - | + | \ | \ | 201.96 |
| Y15 | M | 60 | II | 17 | + | - | - | + | + | - | + | T2N0M0 | 10×10×10 | 274.34 |
| Y17 | M | 73 | II | 29 | + | - | - | + | + | - | + | T3N0M0 | 6×5.5 | 75.62 |
| Y21 | M | 39 | II | 220300 | + | - | + | - | + | - | + | T4N0M0 | 4×4 | 139.18 |
| Y23 | M | 59 | I | 3 | + | - | - | + | + | - | + | T2N0M0 | 7×3.5 | 96.19 |
| Y25 | F | 46 | II | 34 | + | - | - | + | + | - | + | T2N0M0 | 12×7 | 131.39 |
| Y26 | M | 40 | \ | 286 | + | - | - | + | + | - | + | T3N0M0 | 4×4 | 171.72 |
| Y32 | M | 52 | II | 2 | + | - | - | + | + | \ | + | T2N0M0 | 5×5 | 534.52 |
| Y35 | M | 35 | II | 93 | + | - | - | + | + | - | + | T3N0M0 | 16×16 | 225.94 |
| Y36 | F | 73 | II | 32 | + | - | - | + | + | - | + | T2N0M0 | 6×6 | 94.29 |
| Y4 | M | 43 | II | 157800 | + | + | - | + | + | - | + | T3N0M0 | 10×10 | 239.06 |
| Y40 | M | 67 | I | 19 | + | - | + | - | + | - | + | T2N0M0 | 4×3.6 | 85.21 |
| Y41 | M | 59 | \ | 2 | + | - | - | + | + | - | + | T2N0M0 | 5×5 | 461.44 |
| Y43 | M | 50 | II | 3 | + | - | + | - | + | - | + | T3N0M0 | 2.2×2 | 130.42 |
| Y44 | M | 37 | II | 6 | + | - | - | - | + | - | + | T2N0M0 | 15×15 | 202.70 |
| Y46 | M | 37 | II | 205100 | + | - | + | - | + | - | + | T3N0M0 | 15×10 | 358.39 |
| Y49 | M | 39 | I | 449 | + | - | - | + | + | - | + | T2N0M0 | 5×6 | 182.44 |
| Y55 | M | 54 | I | 9 | + | - | - | + | + | - | + | T2N0M0 | 5×5 | 107.84 |
| Y56 | M | 61 | II | 1567 | + | - | - | + | + | - | + | T2N0M0 | 8×8 | 251.53 |
| Y57 | M | 42 | \ | 48 | + | - | - | + | + | - | + | T2N0M0 | 3×3 | 337.02 |
| Y6 | M | 37 | \ | 3890 | + | - | - | + | + | - | + | \ | \ | 461.09 |
| Y60 | F | 49 | II | 1269 | + | - | + | - | + | - | + | T2N0M0 | 6×7 | 137.00 |
| Y64 | M | 62 | II | 29 | + | - | - | + | + | - | + | T2N0M0 | 3×3× | 387.49 |
| Y66 | M | 54 | II | 2757 | + | - | - | + | + | - | + | T3N0M0 | 9×9 | 211.09 |
| Y67 | M | 49 | II | 8721 | + | - | - | + | + | - | + | T4N0M0 | 13×12×7 | 256.28 |
| Y69 | F | 35 | II | 11040 | + | - | + | - | + | - | + | T2N0M0 | 16×16×16 | 254.27 |
| Y72 | M | 61 | \ | 10 | + | - | + | - | + | - | + | T2N0M0 | 3×3 | 163.32 |
| Y74 | F | 51 | I | 3 | + | - | + | - | + | - | + | T2N0M0 | 5.5×5.5 | 167.20 |
| Y75 | M | 54 | II | 6 | + | - | - | + | + | - | + | T1N0M0 | 1.5×1 | 452.66 |
| Y77 | F | 40 | II | 163 | + | - | - | + | + | - | + | T3N0M0 | 1×1 | 429.63 |
| Y84 | M | 51 | I | 1 | + | - | - | - | + | - | + | T2N0M0 | 3×3 | 122.20 |
| Y85 | M | 53 | II | 2531 | + | - | - | + | + | - | + | \ | 6×4.5×3.5 | 206.21 |
| Y87 | M | 71 | II | 4 | + | - | + | - | + | - | - | T1N0M0 | 4.6×7 | 94.80 |
| Y88 | M | 45 | II | 912 | + | - | - | + | + | - | + | T1N0M0 | 6×7 | 210.43 |
| Y91 | M | 37 | \ | 9787 | + | - | - | + | + | - | + | T1N0M0 | 15×15 | 25.21 |
| Y92 | M | 51 | II | 2 | + | - | - | + | + | - | + | T3N0M0 | 13×11×8 | 717.23 |
| Y95 | M | 37 | II | 5 | + | - | - | + | + | - | - | T2N0M0 | 2×1.4 | 69.05 |
| Y96 | F | 56 | \ | 42971 | + | - | - | + | + | - | + | T4N0M0 | 5.3×5.1 | 522.79 |
| Y98 | M | 61 | II | 2 | + | - | - | + | + | - | + | T1N0M0 | 1.5×1.2 | 311.81 |

**Figure S1.** Up-regulated proteins which are expressed in immune organs or cells.


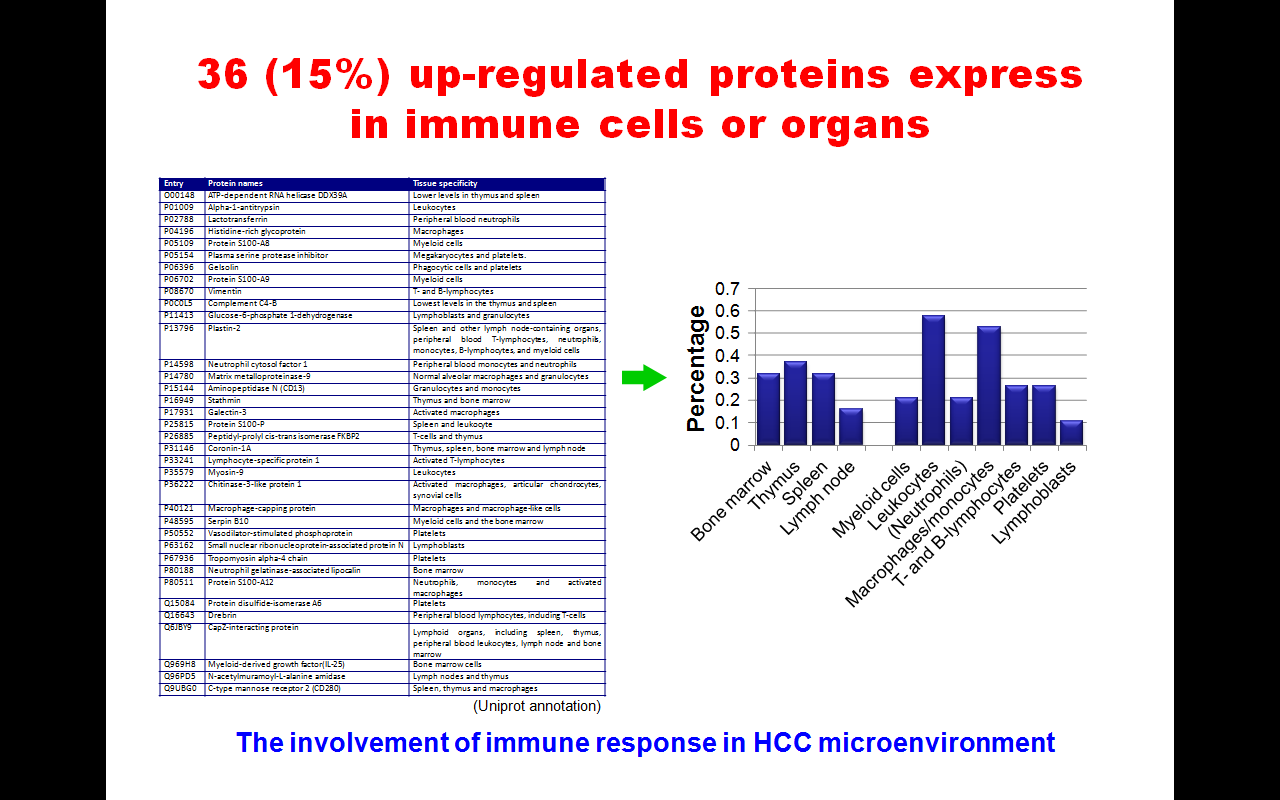

Supplement: Supplementary Information [file srep26499-s1.doc]
